# Crosstalk between gut microbiota-associated metabolites and adaptive immunity in cholecystitis: a conceptual mechanistic framework and potential therapeutic implications

**DOI:** 10.3389/fmicb.2026.1972466

**Published:** 2026-10-06

**Authors:** Xinqi Wang, Tao Zhang, Zhetan Ren, Baozhao Ju

**Affiliations:** 1 Liaoning University of Traditional Chinese Medicine, Shenyang, China; 2 Second Affiliated Hospital, Liaoning University of Traditional Chinese Medicine, Shenyang, China; 3 Department of General Surgery, Beijing Shijitan Hospital, Capital Medical University, Beijing, China

**Keywords:** adaptive immunity, bile acids, cholecystitis, gut microbiota, immunometabolism, microbiota-associated metabolites

## Abstract

The pathogenesis of cholecystitis involves the complex interplay of bile stasis, microbial dysbiosis, metabolic disturbances, and immune responses. Gut microbiota-associated metabolites can modulate adaptive immune components, including T helper 17 (Th17) cells, regulatory T (Treg) cells, CD8^+^ T cells, and B cells, through receptor-mediated signaling, immunometabolic reprogramming, and epigenetic regulation. Conversely, T cell-mediated immune selection and B cell-IgA responses can reshape microbial community structure and metabolic activity, thereby establishing a bidirectional microbiota-immune regulatory network. The immunological effects of metabolic signals, including secondary bile acids, short-chain fatty acids, tryptophan-related metabolites, polyamines, and lactate, are highly context dependent and may vary according to metabolite concentration, target cell type, receptor engagement, and the local tissue microenvironment. Importantly, direct mechanistic evidence specific to cholecystitis remains limited. Most of the mechanistic pathways discussed in this review are derived from studies of cholelithiasis, cholestasis, and other hepatobiliary or inflammatory disorders; therefore, several proposed links between microbial metabolism and adaptive immunity should currently be regarded as conceptual extrapolations rather than established mechanisms in cholecystitis. Focusing on this bidirectional crosstalk, we discuss its potential roles across distinct pathological settings, including gallstone-associated disease, acute inflammation, and chronic inflammation with tissue remodeling. We further summarize candidate therapeutic strategies targeting microbial ecology, bile acid metabolism, specific metabolic pathways, and adaptive immune responses. This conceptual framework may provide a basis for future mechanistic studies and facilitate the development of more precise therapeutic approaches for cholecystitis.

## 1 Introduction

Cholecystitis can be classified along distinct clinical and etiological dimensions, including acute vs. chronic presentations and calculous vs. acalculous forms. Its pathogenesis involves multiple factors, including cystic duct obstruction, bile stasis, ischemic injury of the gallbladder wall, bacterial infection, and local inflammatory responses (Mencarini et al., 2024; Luan and Wang, 2025; Fugazzola et al., 2024), with cholelithiasis representing one of the most common precipitating factors. In recent years, microbiome and metabolomic studies have shown that gallstone formation is accompanied by alterations in gut microbial composition and metabolic profiles (Chang et al., 2024), while the microbial composition of the gallbladder is also closely associated with host bile acid metabolism (Zhang X. et al., 2024). It should be emphasized that the gut microbiota and the gallbladder/biliary tract microbiota represent distinct ecological niches. Accordingly, throughout this review, discussions of microbial metabolism and adaptive immune regulation primarily refer to the gut microbiota, whereas studies specifically addressing microorganisms in bile, the gallbladder, or the biliary tract are explicitly described as involving the gallbladder or biliary microbiota.

The gut microbiota and its metabolic functions have emerged as an important area of investigation in cholelithiasis and related hepatobiliary disorders. Mendelian randomization studies have suggested potential causal associations between specific gut microbial taxa, circulating metabolites, and the risk of gallstone disease (Hu et al., 2024). In this review, we use the broad term microbiota-associated metabolites to encompass metabolites directly produced by microorganisms, host-derived metabolites that undergo further microbial transformation or modification, and metabolites primarily generated through host metabolic pathways but substantially influenced by the gut microbiota. Representative metabolic signals include secondary bile acids, short-chain fatty acids (SCFAs), tryptophan-related metabolites, and polyamines (Zeng et al., 2025). Secondary bile acids, for example, are generated through microbial transformation of host-derived primary bile acids (Ridlon and Gaskins, 2024). By contrast, kynurenine is predominantly produced through host tryptophan metabolism, although its abundance and metabolic flux can be modulated by the gut microbiota and inflammatory conditions (Tan et al., 2025).

Adaptive immunity may represent an important effector interface linking microbiota-associated metabolic disturbances to gallbladder inflammation. Gut microorganisms and their metabolic signals can regulate multiple adaptive immune compartments, including CD4^+^ T cells, CD8^+^ T cells, and B cells (Heidari et al., 2024). In particular, SCFAs, microbially modified bile acids, and aromatic metabolites can influence the Th17/Treg balance and effector T-cell function (Li et al., 2025; Cong et al., 2024; Antonini Cencicchio et al., 2025; Kim, 2023; Chen et al., 2025). Conversely, T cell–mediated immune selection and B cell–IgA responses can reshape microbial colonization and ecological competition within the intestine (Zheng et al., 2020; Tejedor Vaquero et al., 2024), thereby establishing a bidirectional metabolic–immune interaction. Importantly, the immunological effects of these metabolites are strongly context dependent and may vary according to metabolite identity and concentration, target cell type, receptor engagement, and the local tissue microenvironment.

Direct mechanistic studies specifically examining microbiota-associated metabolite-adaptive immunity interactions in cholecystitis remain limited. The evidence synthesized in this review therefore spans several levels, including studies of human cholecystitis and gallbladder tissues, studies of cholelithiasis, cholestasis, and other related hepatobiliary disorders, as well as mechanistic investigations conducted in other diseases or experimental models, including intestinal inflammation, cancer, and autoimmune disorders. Relationships that have not yet been directly validated in cholecystitis are discussed only as conceptual extrapolations. The review focuses primarily on gallstone-associated disease, particularly calculous cholecystitis. Acalculous cholecystitis, which has distinct etiological and pathophysiological features, is not a primary focus of this review. Acute and chronic cholecystitis are considered distinct clinical and pathological contexts rather than obligatorily sequential stages of a single disease process.

Against this background, this review focuses on the bidirectional crosstalk between microbiota-associated metabolites and adaptive immunity. We summarize the major mechanisms through which bile acids, SCFAs, tryptophan-related metabolites, polyamines, and lactate regulate T- and B-cell responses, and integrate evidence from gallbladder disease and related hepatobiliary conditions to discuss their potential relevance to cholecystitis. On this basis, we propose a conceptual mechanistic framework and highlight potential directions for future therapeutic intervention.

## 2 Bidirectional crosstalk between microbiota-associated metabolites and adaptive immunity

### 2.1 Regulation of adaptive immunity by microbiota-associated metabolites

Microbiota-associated metabolites can influence T-cell differentiation and effector function through receptor-mediated signaling, immunometabolic reprogramming, and epigenetic regulation. Among these metabolites, bile acid derivatives are particularly relevant to hepatobiliary diseases. After primary bile acids enter the intestine, they undergo microbial oxidation, dehydroxylation, epimerization, and other transformations, generating a diverse range of immunologically active secondary bile acids and their derivatives. 3-OxoLCA can inhibit RORγt transcriptional activity, thereby restricting Th17-cell differentiation and IL-17-associated responses (Paik et al., 2022; Hang et al., 2019). By contrast, isoalloLCA and other specific bile acid derivatives can promote Foxp3 expression and Treg differentiation through mechanisms involving nuclear receptor signaling, mitochondrial reactive oxygen species, and related transcriptional programs (Li et al., 2021; Campbell et al., 2020). Additional derivatives generated from further metabolism of lithocholic acid (LCA) can regulate Th17/Treg differentiation through RORγt-, vitamin D receptor (VDR)-, and other nuclear receptor-associated pathways (Lindner et al., 2024; Xu et al., 2025). Secondary bile acids such as deoxycholic acid (DCA) can also modulate CD4^+^ T-cell activation and T-cell-driven inflammatory responses (Wang et al., 2022; Li et al., 2023). Collectively, these findings indicate that the immunological effects of bile acids are determined by their specific molecular structures, receptor engagement, and target cell populations rather than following a uniform directional pattern.

Short-chain fatty acids (SCFAs) are another major class of metabolic mediators involved in adaptive immune regulation. Butyrate can promote Foxp3^+^ Treg differentiation and functional maintenance through histone deacetylase (HDAC) inhibition and pathways related to mTOR signaling, autophagy, and oxidative metabolism (He et al., 2024; Park et al., 2015). Propionate can influence CD4^+^ T-cell differentiation through FFAR2/FFAR3 signaling, HDAC modulation, and metabolic reprogramming, and under specific conditions may restrain excessive Th1/Th17 responses or favor regulatory immune phenotypes (Han et al., 2024; González-Brito and Uribe-Herranz, 2023). Acetate, in turn, participates in the metabolic adaptation of activated T cells through pathways such as ACSS2–acetyl-CoA metabolism and can modulate the proliferation and effector functions of both CD4^+^ and CD8^+^ T cells (Miller et al., 2023; Huang et al., 2023). The immunological effects of SCFAs are therefore shaped by metabolite concentration, T-cell activation state, nutrient availability, and the host immune context, and should not be reduced to a simple unidirectional immunosuppressive effect.

Tryptophan-related metabolites primarily regulate adaptive immunity through the aryl hydrocarbon receptor (AHR) and associated metabolic-sensing pathways. Microbiota-derived indole metabolites such as indole-3-aldehyde (IAld) can act as AHR ligands and influence T-cell-associated transcriptional programs and immune homeostasis (Bender et al., 2023; Bahman et al., 2024). Other metabolites, including indole-3-lactic acid (ILA), can also regulate CD4^+^ T-cell differentiation through AHR-dependent and related immunometabolic signaling pathways (Cervantes-Barragan et al., 2017; Alexander et al., 2024). Kynurenine and its downstream metabolites can modulate effector T-cell infiltration and function through AHR activation and immunoregulatory programs (Liu et al., 2026; Lin et al., 2025). Importantly, kynurenine is generated predominantly through host tryptophan metabolism, although its production and downstream metabolic flux are jointly influenced by the gut microbiota, inflammatory state, and host metabolism. Moreover, different AHR ligands may exert distinct or even divergent effects depending on ligand identity, target cell type, and disease context.

Polyamines and lactate also contribute to the regulation of adaptive immunity. Spermidine can modulate Atg5-dependent autophagy, mTOR signaling, and T-cell receptor-associated metabolic programs, thereby influencing the functions of Foxp3^+^ Treg cells and effector T cells (Carriche et al., 2021; Wang T. et al., 2024). These effects are likewise dependent on metabolite concentration and cellular context. Lactate can serve not only as a metabolic substrate for T cells but also as a signaling and epigenetic regulator capable of reshaping CD4^+^ and CD8^+^ T-cell differentiation and effector states through metabolic reprogramming, protein lactylation, and HDAC–H3K27ac–TCF1-associated epigenetic mechanisms (Feng et al., 2022; Raychaudhuri et al., 2024). Its immunological effects are further modulated by local lactate concentrations, extracellular pH, nutrient availability, and cellular metabolic demands. Lactate therefore cannot be uniformly classified as either a pro-inflammatory or an immunosuppressive metabolite.

Overall, bile acids, SCFAs, tryptophan-related metabolites, polyamines, and lactate are all capable of regulating adaptive immune responses, but the direction and magnitude of their effects are strongly context dependent. Their biological consequences are jointly determined by metabolite identity and concentration, receptor usage, target cell type, and the local tissue microenvironment. The divergent, and in some cases apparently opposing, effects reported across studies underscore the importance of interpreting these metabolic signals within their specific biological and disease contexts.

### 2.2 Reciprocal regulation of gut microbial metabolism by adaptive immunity

While adaptive immunity responds to microbiota-associated metabolic signals, it can also reciprocally shape microbial metabolic functions by altering microbial colonization, spatial organization, and ecological competition. At present, direct evidence for this reciprocal regulation is derived primarily from animal models and studies conducted in disease contexts other than cholecystitis. Loss of adaptive immunity can markedly alter the biogeographical distribution of microbial communities along different segments of the gastrointestinal tract, while T-cell deficiency can simultaneously reshape gut microbial composition and the local metabolomic landscape (Gu et al., 2022; Caspani et al., 2022). Age-related deterioration of adaptive immunity can likewise induce functional remodeling of the microbiota, including reduced microbial capacity for vitamin B6 biosynthesis and salvage pathways, accompanied by decreased circulating vitamin B6 levels in the host (Stahl et al., 2026). These observations suggest that the state of adaptive immunity can act upstream to modify microbial function and metabolic output.

B cell- and plasma cell-mediated IgA responses represent a major mechanism underlying this reciprocal regulation. IgA deficiency can lead to dysbiosis of the ileal microbiota, accompanied by enhanced mucosal T-cell activation and inflammation (Nagaishi et al., 2022). Secretory IgA selectively recognizes and coats specific microorganisms, thereby regulating their access to the mucosal surface, spatial distribution, and ecological competitiveness. Different commensal organisms can elicit distinct IgA responses, and T cell-dependent IgA selection contributes to the establishment of stable microbiota–antibody relationships (Zhang S. et al., 2023). Alterations in maternal secretory IgA can also influence early-life microbial assembly and patterns of host–microbe contact in offspring (Donald et al., 2024), whereas HIF-1α-dependent metabolic programs in B cells further regulate IgA production and mucosal homeostasis (Meng et al., 2025). Because SCFA production, bile acid transformation, and tryptophan metabolism depend on microbial communities with distinct functional capacities, IgA-mediated microbial selection may in turn modify the output of these metabolic pathways.

T cells appear to shape microbial metabolic niches largely through cytokine–epithelial signaling. T cell-associated immune signals such as IL-22 can act on the intestinal epithelium to modify antimicrobial peptide production, mucus properties, and glycosylation programs, thereby influencing microbial composition and function. Experimental studies have shown that IL-22 signaling can enhance microbial tryptophan metabolism, increase the production of indole derivatives, and augment AHR activity (Mar et al., 2023). IL-22–STAT3-associated epithelial repair pathways can also improve barrier integrity while simultaneously reshaping the microbial environment (Zhang P. et al., 2024). Alterations in T-cell immune programs may themselves be accompanied by changes in microbial metabolic output. For example, T-bet deficiency has been associated with restructuring of specific microbial communities and increased fecal butyrate levels, and the corresponding SCFA phenotype can be partially transferred through microbiota transplantation (Stolarczyk et al., 2024). Similarly, in T-cell-deficient models, microbial developmental trajectories and the gastrointestinal metabolome are altered in parallel (Girdhar et al., 2023). Together, these findings indicate that T cells can influence microbial metabolic functions by modifying the epithelial environment, inflammatory state, and ecological niche.

The gut–liver–biliary axis may provide an additional intermediate pathway through which adaptive immunity influences microbial bile acid metabolism. Cholestasis alters both the composition and concentration of bile acids entering the intestinal lumen and is accompanied by changes in gut microbial composition and bile acid-transforming capacity (Yang et al., 2024). In turn, shifts in the intestinal bile acid environment can select for microorganisms possessing bile salt hydrolases, hydroxysteroid dehydrogenases, and 7α-dehydroxylation activity, thereby modifying the balance between primary and secondary bile acids (Tang et al., 2023). Accordingly, when persistent T- or B-cell-driven inflammation alters bile flow or bile acid composition, it could theoretically modify the selective bile acid environment experienced by the gut microbiota and thereby reshape the generation of secondary bile acids and their bioactive derivatives. At present, however, this proposed adaptive immunity–bile acid environment–microbial bile acid metabolism axis is supported mainly by evidence from related hepatobiliary disorders and mechanistic studies rather than by direct studies in cholecystitis.

Adaptive immune dysregulation may also alter microbial metabolic functions by modifying epithelial metabolism, the mucus environment, and nutrient substrate availability. Changes in host immune status can induce spatial redistribution of the microbiota (He et al., 2025) and alter competition between obligate and facultative anaerobes, with downstream consequences for carbohydrate fermentation and amino acid metabolism. Integrated microbiome–metabolome studies have further shown that abnormalities in adaptive immunity are frequently accompanied by changes in SCFAs, aromatic amino acid metabolites, and other microbiota-associated metabolites (Nakajima et al., 2018; Joglekar et al., 2019; Longoria et al., 2026). Notably, antigen-specific mucosal antibodies can modulate both microbial and host metabolic functions, including bile acid-related metabolic outputs, even in the absence of major changes in overall community composition (Vaz et al., 2026). This finding suggests that adaptive immunity can regulate microbial metabolism not only through shifts in community structure but also through functional remodeling of existing microbial populations.

Overall, adaptive immunity can reciprocally regulate microbial composition and metabolic function through multiple mechanisms, including IgA-mediated selection, T cell–epithelial signaling, modulation of the bile acid environment, and remodeling of nutritional niches. These pathways provide a mechanistic basis for bidirectional feedback between microbiota-associated metabolites and adaptive immunity. However, the specific configuration and relative contribution of these regulatory circuits in cholecystitis remain to be defined through integrated analyses of bile, gallbladder tissue, and the intestinal microbiome.

## 3 Potential roles of microbiota-associated metabolite–adaptive immunity crosstalk in cholecystitis

To date, no longitudinal clinical or mechanistic study has demonstrated a fixed “initiation–amplification–chronic remodeling” sequence in cholecystitis driven by dysregulated microbiota-associated metabolism and adaptive immunity. Acute and chronic cholecystitis should likewise not be regarded simply as sequential stages of a single disease process. Accordingly, this section is organized around distinct clinical and pathological contexts. Evidence from cholecystitis and gallstone-associated studies is integrated with findings from cholestatic and other related hepatobiliary disorders, as well as cross-disease immunometabolic research, to discuss the potential links between microbiota-associated metabolites and adaptive immunity in cholecystitis. These context-dependent and nonsequential interactions are summarized schematically in Figure 1.

**Figure 1:**
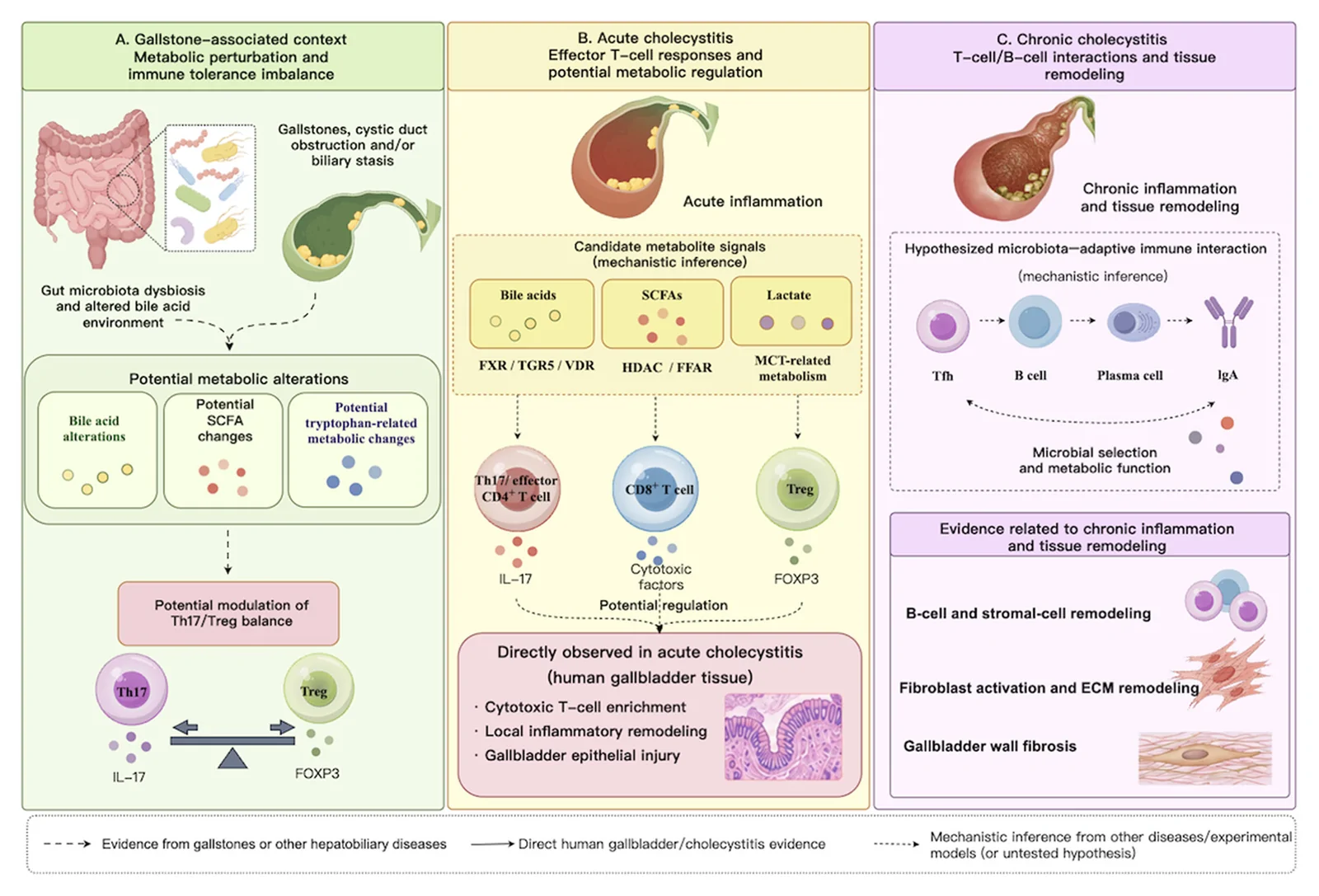
Context-dependent crosstalk between microbiota-associated metabolites and adaptive immunity across cholecystitis-related pathological contexts. The schematic integrates current evidence on microbiota-associated metabolite–adaptive immune interactions across three distinct, non-sequential clinical and pathological contexts. **(A)** Gallstone-associated context: gallstones, cystic duct obstruction, biliary stasis, gut microbial dysbiosis, and alterations in the bile acid environment may be accompanied by changes in bile acids, SCFAs, and tryptophan-related metabolites, which could potentially influence the Th17/Treg balance. **(B)** Acute cholecystitis: candidate metabolic signals, including bile acids, SCFAs, and lactate, may modulate Th17/effector CD4^+^ T cells, CD8^+^ T cells, and Treg cells through receptor-dependent and immunometabolic pathways involving FXR/TGR5/VDR, HDAC/FFAR signaling, and MCT-related lactate metabolism. Human gallbladder studies directly support cytotoxic T-cell enrichment, local inflammatory remodeling, and epithelial injury in acute cholecystitis, whereas the upstream metabolite–T-cell links remain largely mechanistic inferences. **(C)** Chronic cholecystitis: Tfh–B-cell–plasma-cell–IgA interactions may reciprocally influence microbial selection and metabolic function, while B-cell/stromal remodeling, fibroblast activation, extracellular matrix remodeling, and gallbladder wall fibrosis are supported to varying degrees by direct gallbladder and related hepatobiliary evidence. Solid arrows indicate direct evidence from human gallbladder/cholecystitis studies; dashed arrows indicate evidence from gallstone disease or other hepatobiliary disorders; dotted arrows indicate mechanistic inference from other diseases or experimental models, or untested hypotheses. Acute and chronic cholecystitis are presented as distinct clinical/pathological contexts rather than consecutive disease stages, and the immunological effects of metabolites should be interpreted as context dependent.

### 3.1 Gallstone-associated metabolic perturbations and disruption of adaptive immune tolerance

Gallstone formation, cystic duct obstruction, and bile stasis constitute major pathological backgrounds of calculous cholecystitis and can be accompanied by disturbances in the enterohepatic circulation of bile acids and alterations in the gut microbiota. Animal studies have shown that the gut microbiota can promote cholesterol gallstone formation by modifying bile acid composition and biliary cholesterol secretion. Furthermore, single-cell transcriptomic analyses of the gallbladder in cholesterol gallstone models have revealed extensive remodeling of both epithelial and non-epithelial cell populations, accompanied by epithelial proliferation, immune-cell infiltration, angiogenesis, and extracellular matrix-associated changes (Hu H. et al., 2022; Liang et al., 2021). These findings suggest that gallstone-associated alterations in the biliary environment may not only directly affect local gallbladder tissues but may also influence microbial metabolic functions by changing the bile acid substrates and selective pressures reaching the intestine.

Changes in the bile acid pool may, in turn, influence adaptive immune regulation. Studies of intestinal inflammation, autoimmune disease, and related hepatobiliary disorders have shown that different microbially modified bile acids can exert distinct, and in some cases opposing, effects on Th17 and Treg differentiation (Biagioli et al., 2024; Song et al., 2020). The balance between bile acid conjugation and deconjugation can also modulate the severity of intestinal inflammation, with these immunological effects being closely linked to microbial enzymatic activity and overall bile acid pool composition (Fu et al., 2025). In cholestatic models, FXR signaling, intestinal barrier integrity, and microbial community structure may also change in parallel, with downstream consequences for bile acid metabolism (Xie et al., 2025). Thus, alterations in the bile acid environment caused by gallstones and cholestasis provide a biologically plausible basis for disrupted adaptive immune tolerance. However, the exposure levels of specific bile acid metabolites, such as 3-oxoLCA and isoalloLCA, in bile or gallbladder tissue from patients with cholecystitis, as well as their local effects on T-cell responses, remain to be established.

Beyond bile acids, SCFAs and tryptophan-related metabolites may also contribute to the link between gallstone-associated metabolic disturbances and adaptive immunity. Experiments using human T cells have shown that acetate, propionate, and butyrate can promote the differentiation and suppressive function of inducible Treg cells while altering Foxp3-associated transcriptional programs (Hu M. et al., 2022). Accordingly, if gallstone-associated changes in the biliary environment and intestinal ecology alter SCFA-producing microbial populations, the resulting changes in metabolic output could potentially affect Treg homeostasis. Similarly, alterations in tryptophan metabolism and AHR ligand availability may influence Th17/Treg differentiation. Metabolomic and functional studies in other inflammatory diseases have demonstrated that combinations of gut-derived metabolites can reshape the inflammatory and pathogenic states of Th17 cells (Rebeaud et al., 2025).

Taken together, alterations in bile acids, SCFAs, and tryptophan-related metabolites provide a mechanistic basis for linking gallstone-associated metabolic disturbances with changes in adaptive immune tolerance. Nevertheless, current direct evidence remains concentrated on gallstone formation, alterations in the biliary environment, and remodeling of gallbladder tissues, whereas the specific metabolite–T-cell interactions are supported largely by cross-disease mechanistic studies. At present, this relationship is therefore best regarded as a conceptual mechanistic hypothesis in the context of calculous cholecystitis rather than an established disease-specific pathway.

### 3.2 Effector T-cell responses and potential metabolic regulation in acute cholecystitis

Acute cholecystitis is characterized by prominent local inflammatory-cell infiltration and tissue injury, yet direct causal evidence linking microbiota-associated metabolic signals to effector T-cell responses remains limited. Mechanistic studies conducted in other disease contexts nevertheless provide a biological basis for such a possibility. For example, 24-nor-ursodeoxycholic acid has been shown to restrict glutamine metabolism and pathogenicity in Th17 cells while increasing the proportion of Treg cells (Zhu et al., 2025). In fibrosing cholangiopathies, bile acid retention can impair Treg function and induce a Th17-like transcriptional program through S1P receptor–STAT3-associated signaling (Kudira et al., 2025). In addition, a clinical study in type 1 diabetes showed that reconstruction of the gut microbiota increased levels of secondary bile acids, including LCA and isoLCA, in parallel with reductions in Th17 cells, IL-17A, and systemic inflammation (Liu et al., 2025). Collectively, these findings indicate that changes in bile acid composition can modulate the Th17/Treg balance; however, their specific contribution to local T-cell responses in acute cholecystitis has not yet been directly established.

The immunological effects of SCFAs are likewise strongly dependent on host context. Clinical intervention studies have shown that propionate supplementation can expand peripheral Treg populations in patients with end-stage renal disease, whereas a comparable response was not observed in kidney transplant recipients receiving immunosuppressive therapy (Anft et al., 2024). This discrepancy suggests that even if acute cholecystitis is accompanied by changes in SCFA abundance or composition, their actual effects on effector T cells and Treg cells may still be jointly determined by baseline immune status, treatment exposure, and the local metabolic environment.

Lactate further illustrates the bidirectional influence of the local metabolic milieu on T-cell function. Activated T cells can take up lactate and use it as a metabolic substrate, and lactate exposure can substantially reshape the transcriptional and metabolic programs of CD8^+^ T cells (Barbieri et al., 2023). At the same time, lactate-rich environments can promote metabolic adaptation and PD-1 expression in Treg cells, thereby supporting their survival and functional maintenance within highly glycolytic tissues (Kumagai et al., 2022; Watson et al., 2021). Sustained lactate uptake can also promote the development of terminally exhausted CD8^+^ T cells, whereas blockade of MCT11-mediated lactate uptake can partially restore their effector function (Peralta et al., 2024). Thus, the effects of lactate on T cells depend on its concentration, the specific T-cell subset involved, and the local nutritional and metabolic context, and cannot be assigned a fixed pro-inflammatory or anti-inflammatory role.

Compared with these cross-disease metabolic mechanisms, studies of human gallbladder tissue provide more direct evidence of local immune remodeling. Single-cell analyses have shown marked enrichment of pro-inflammatory myeloid cells and cytotoxic T cells in acute cholecystitis, accompanied by disruption of gallbladder epithelial architecture (Vlasenko et al., 2026). Immunohistochemical studies have further identified differences in the expression of inflammatory mediators, including IL-2R, IL-6, TNF-α, and substance P, among acute cholecystitis, chronic cholecystitis, and gallstone-bearing gallbladders with histologically normal tissue (Psaltis et al., 2024). These findings support the presence of substantial local immune remodeling in acute cholecystitis but do not establish that such changes are directly driven by specific alterations in bile acids, SCFAs, or lactate.

Evidence from related hepatobiliary disorders further suggests that the metabolic environment and local inflammation may influence one another. In bile duct ligation models, reduced delivery of bile acids to the intestine markedly alters gut microbial composition and function and is accompanied by changes in circulating inflammatory mediators and the hepatic bile acid profile (Leung et al., 2023). Accordingly, in acute cholecystitis, microbiota-associated metabolites are more appropriately considered potential modulators of effector T-cell responses rather than established drivers of inflammation.

### 3.3 T/B-cell interactions and tissue remodeling in chronic cholecystitis

Chronic cholecystitis is commonly accompanied by persistent inflammation, alterations in immune-cell composition, and tissue fibrosis. Single-cell studies of human gallbladder tissue have shown that, compared with the prominent pro-inflammatory myeloid and cytotoxic T-cell responses observed during acute inflammation, chronic gallstone-associated tissues exhibit more pronounced intercellular communication among macrophages, B cells, and fibroblasts, together with substantial fibrotic and extracellular matrix remodeling (Vlasenko et al., 2026). Studies of gallbladder tissue from pediatric patients with chronic calculous cholecystitis have further identified dysregulation of tissue repair- and growth-associated signals, including SHH, IHH, IGF1R, and HOXB3 (Derbeneva et al., 2025). These direct observations in gallbladder tissue suggest that chronic cholecystitis is characterized by an immune–stromal microenvironment distinct from that of acute inflammation, although its causal relationship with alterations in microbial metabolism remains unclear.

B cells and plasma cells may participate in this crosstalk through IgA-mediated responses. Current mechanistic evidence is derived primarily from studies of the intestine and other mucosal immune systems. IgA can selectively recognize and constrain intestinal microorganisms with relatively high inflammatory potential, and experimental manipulation of IgA responses can alter the growth and ecological organization of targeted microbial populations (Takahashi et al., 2024). IgA-seq studies have further demonstrated that different intestinal microorganisms exhibit distinct patterns of IgA coating, allowing the identification of microbial populations with greater immunostimulatory potential (van Gogh et al., 2024). Persistent inflammation can also remodel the IgA antibody repertoire and patterns of microbial reactivity, thereby altering mucosal antibody-mediated selective pressure (Melcher et al., 2025). Accordingly, if B-cell or plasma-cell responses remain persistently altered in chronic cholecystitis, IgA-mediated microbial selection could theoretically influence metabolic functions related to bile acids, SCFAs, and aromatic amino acids. However, direct evidence for such a mechanism in cholecystitis is currently lacking.

Coordination between T and B cells may further reinforce this bidirectional regulation. Studies of the intestinal mucosa have shown that dietary and microbial signals can induce commensal-specific T follicular helper (Tfh) cells and IgA responses, suggesting that T cells can shape microbial ecology by regulating B-cell antibody responses (Hattori-Muroi et al., 2026). Microbiota-derived butyrate can also modulate Tfh–B-cell interactions and mucosal IgA production (Ko et al., 2026). These findings indicate that microbial metabolites, T-cell help, and B-cell antibody responses can form an interconnected regulatory network. Nevertheless, whether a Tfh–B-cell–IgA axis contributes to local immune and metabolic homeostasis in chronic cholecystitis remains an untested mechanistic hypothesis.

The bile acid environment associated with chronic inflammation may, in turn, reshape the metabolic ecology of the intestinal microbiota. Bile acids themselves impose selective pressure on intestinal microorganisms, while specific bile acids can exert distal metabolic effects by modifying the local environment and microbial composition of the small intestine (Chen et al., 2024). Bacterial bile salt hydrolases can additionally promote the formation of novel microbially conjugated bile acids, thereby expanding the structural and functional diversity of the bile acid pool (Rimal et al., 2024). Persistent changes in bile flow and the composition of available bile acid substrates may therefore alter the bile acid-transforming capacity of the gut microbiota, although the magnitude and direction of this effect in chronic cholecystitis remain to be determined.

At the tissue level, persistent immune dysregulation may ultimately converge with remodeling of the gallbladder wall. Studies of related hepatobiliary disorders have shown that Clec3b^+^ portal fibroblasts rapidly expand after biliary injury and become a major source of portal myofibroblasts. Their activation is regulated by the KLF4–periostin axis and directly contributes to biliary-associated portal fibrosis (Lei et al., 2026; Wang et al., 2025). Meanwhile, chronically inflamed gallstone-associated gallbladder tissues themselves display prominent stromal and fibrotic remodeling (Vlasenko et al., 2026; Derbeneva et al., 2025). Together, these observations provide biological plausibility for a model in which T/B-cell immune dysregulation, alterations in the biliary environment, and fibroblast activation may collectively contribute to chronic remodeling of the gallbladder wall. However, cholecystitis-specific longitudinal evidence linking these individual components into a continuous causal pathway is still lacking.

Overall, chronic cholecystitis can be conceptualized as involving a hypothetical network connecting microbial metabolic function, T- and B-cell adaptive immunity, the biliary environment, and tissue remodeling. This framework integrates direct evidence from gallbladder tissues with findings from related hepatobiliary disorders and cross-disease mechanistic studies. Its primary purpose is to organize the available evidence and generate experimentally testable hypotheses, rather than to define an established natural history or a fixed causal sequence of chronic cholecystitis.

## 4 Potential therapeutic strategies targeting gut microbiota-associated metabolite–adaptive immunity crosstalk

At present, no cholecystitis-specific therapy has been established that directly targets the gut microbiota-associated metabolite–adaptive immunity axis. Clinical management of cholecystitis continues to be guided primarily by disease etiology and severity, as well as the presence of gallstones, biliary obstruction, infection, and related complications. Accordingly, the microbiota-, metabolism-, and immunity-directed approaches discussed below should be regarded as candidate interventions supported by varying levels of evidence rather than established treatments for cholecystitis. Their principal mechanisms, sources of evidence, and major limitations are summarized in Table 1.

**Table 1:** Candidate therapeutic strategies targeting microbiota-associated metabolite–adaptive immune crosstalk relevant to cholecystitis.

Strategy	Key mechanism	Evidence source/model	Evidence status	Hypothesized relevance to cholecystitis	Major limitation	References
Dietary fiber/prebiotics	Modify fermentable substrates and microbial SCFA, bile acid and tryptophan metabolism	Human dietary interventions; preclinical inflammatory models	Human cross-disease and preclinical evidence	May modify gallstone-associated dysbiosis and downstream immune-metabolic signaling	Strongly dependent on baseline microbiota, fiber type and dose; fermentation is not uniformly anti-inflammatory	Wastyk et al., 2021; Holmes et al., 2022; Arifuzzaman et al., 2022
Probiotics/synbiotics	Alter microbial competition, barrier function and metabolite production	Mainly mouse colitis and other inflammatory models	Predominantly preclinical evidence	May correct dysbiosis-associated metabolic abnormalities and indirectly influence adaptive immunity	Marked strain/formulation dependence and variable engraftment; no cholecystitis-specific RCTs	Xu et al., 2022; Lee et al., 2024; Dai et al., 2024
FMT/defined microbial consortia	Restore microbial functional networks and secondary bile acid/SCFA production	Human IBD cohorts; experimental colitis models	Cross-disease human and preclinical evidence	May restore microbial metabolic functions disrupted by dysbiosis	Donor variability, transmission risk and uncertain engraftment; indication in cholecystitis unknown	van der Lelie et al., 2021; Zhou et al., 2023; Lu et al., 2025
Microbial bile acid enzyme targeting	Modulate BSH, 7α-dehydroxylation and related bile acid transformations	In vitro microbial enzyme studies; animal/metabolic models	Mechanistic and preclinical evidence	May reshape exposure to immunologically active bile acid species	Functional redundancy and substrate dependence may cause compensatory metabolic changes	Adhikari et al., 2021; Foley et al., 2021; Guzior et al., 2024; Wahlström et al., 2024
UDCA/norUDCA and bile acid pool modulation	Alter bile acid composition, enterohepatic circulation and immune-metabolic signaling	PSC and other cholestatic hepatobiliary studies; mechanistic models	Clinical evidence in related hepatobiliary disease plus preclinical evidence	Most directly relevant to bile acid-disturbed hepatobiliary environments, but effects on cholecystitis-specific immune crosstalk remain unknown	Efficacy in cholestatic disorders cannot be directly extrapolated to cholecystitis	Zhu et al., 2021; Zhang C. et al., 2025; Fickert et al., 2017
FXR/TGR5/VDR-related modulation	Regulate bile acid synthesis/transport, epithelial homeostasis and inflammatory signaling	IBD, sepsis and hepatobiliary experimental/translational studies	Preclinical/translational evidence	May alter the gut–liver–gallbladder metabolic environment and indirectly affect adaptive immunity	Broad receptor expression limits tissue and immune-cell specificity	Zhao et al., 2025; Reich et al., 2025; Gubatan et al., 2026
SCFA supplementation/targeted delivery	Modulate HDAC, FFAR, autophagy and T-cell immunometabolism	Experimental colitis and targeted-delivery models	Predominantly preclinical evidence	May influence Treg/effector T-cell balance when SCFA availability is altered	Rapid absorption and uncertain tissue exposure; effects are concentration- and context-dependent	Mu et al., 2021; Su et al., 2024; Park et al., 2026
Tryptophan–AHR pathway modulation	Modify ligand-specific AHR signaling in Th17/Treg and other T-cell programs	Human *ex vivo* immune studies; autoimmune/inflammatory models	Cross-disease mechanistic and preclinical evidence	May influence adaptive immunity if tryptophan metabolism is altered	Strong ligand-, dose-, cell- and disease-context dependence; no direct cholecystitis evidence	Liu et al., 2023; Yu et al., 2023; Cao et al., 2024
Polyamine/spermidine modulation	Affect autophagy, TCR signaling and T-cell metabolic adaptation	Autoimmune and tumor immunology models	Preclinical mechanistic evidence	May modify Treg and effector T-cell function	Effects may be regulatory or suppressive depending on dose, cell type and microenvironment	Karacay et al., 2022; Hibino et al., 2023; Kay et al., 2025
Lactate/MCT modulation	Alter lactate uptake and T-cell effector, regulatory, memory and exhaustion programs	Tumor immunology and T-cell metabolic models	Mechanistic/preclinical evidence	May modify T-cell states in a metabolically altered gallbladder microenvironment	MCTs are required for normal T-cell metabolism; effects depend on lactate concentration and pH	Zhou et al., 2022; Lopez et al., 2023; D'Aria et al., 2024
Th17/IL-17 pathway inhibition	Suppress IL-17-associated inflammatory and profibrotic signaling	Other immune-mediated diseases and inflammatory/fibrotic models	Clinical evidence in other diseases plus preclinical evidence	May reduce T-cell-associated inflammatory injury if the pathway is locally active	Does not correct upstream dysbiosis/metabolic abnormalities and may impair mucosal host defense	Zhang C. et al., 2024; Ma et al., 2024; Riani Moreira et al., 2025
Treg-enhancing strategies, including low-dose IL-2	Expand/stabilize Foxp3^+^ Treg and rebalance Th17/Treg responses	RA clinical studies; PBC and other immune-mediated models	Early cross-disease clinical and preclinical hepatobiliary evidence	May strengthen immune regulation in T-cell-dysregulated cholecystitis	Systemic Treg expansion lacks gallbladder specificity and may cause unwanted immunosuppression	Tchitchek et al., 2022; Wang Z. et al., 2024; Yan et al., 2025
Tfh–B-cell–IgA axis modulation	Modify IgA production and microbial coating/selection	Human gut microbiome studies; mucosal and colitis models	Human mechanistic and preclinical evidence	May alter reciprocal B-cell–microbiota–metabolite interactions	Highly antigen- and strain-specific; therapeutic manipulation remains experimental	Hattori-Muroi et al., 2023; Jin et al., 2025; Olm et al., 2025

Bile acid-directed interventions currently have the most direct clinical relevance to hepatobiliary disease. Ursodeoxycholic acid (UDCA), norUDCA, and related bile acid-based interventions have been investigated clinically in several cholestatic hepatobiliary disorders and can modify the inflammatory and metabolic milieu by altering the bile acid pool, enterohepatic circulation, and receptor-mediated signaling. Mechanistic studies have further suggested that norUDCA can modulate T-cell immunometabolism. Strategies targeting bile acid-responsive receptors, including FXR, TGR5, and VDR, may likewise influence bile acid synthesis and transport, epithelial homeostasis, and inflammatory signaling. However, most of this evidence derives from primary sclerosing cholangitis, other cholestatic disorders, and experimental models, and these approaches cannot currently be considered established or effective therapies for cholecystitis.

Strategies aimed at remodeling microbial ecology and function are at a comparatively earlier stage of translation. Dietary fiber, prebiotics, probiotics, synbiotics, fecal microbiota transplantation (FMT), and defined microbial consortia can alter microbial community composition and functional output, with downstream effects on SCFAs, secondary bile acids, and tryptophan-related metabolites. Some of these approaches have entered human studies in other disease settings, but cholecystitis-specific clinical evidence remains lacking. Compared with broad microbiota-directed interventions, targeting microbial enzymes involved in bile acid metabolism, such as bile salt hydrolases (BSHs), 7α-dehydroxylation pathways, and other microbial metabolic enzymes, may provide a more direct means of modulating bile acid transformation and the composition of the bile acid pool. Nevertheless, such strategies remain predominantly at the mechanistic and preclinical stages.

Interventions targeting specific metabolic pathways require even greater caution. SCFAs, tryptophan–AHR signaling, polyamine metabolism, and lactate metabolism can all influence Treg, Th17, and CD8^+^ T-cell responses, but their effects are determined jointly by metabolite identity and concentration, receptor expression, target cell type, and the local tissue microenvironment. These strategies may therefore be more appropriately developed as phenotype-guided interventions directed at clearly defined metabolic abnormalities and immune states, rather than being conceptualized simply as supplementation of “beneficial” metabolites or inhibition of “harmful” metabolites. Current evidence is derived largely from *in vitro* experiments, animal studies, and other disease contexts, and the tissue exposure, effective dose ranges, and safety profiles required for application in cholecystitis remain to be established.

Downstream adaptive immune pathways also provide potential therapeutic targets, including inhibition of the Th17/IL-17 axis, enhancement of Treg responses, and modulation of the Tfh–B-cell–IgA axis. IL-17 blockade and low-dose IL-2 therapy have shown clinical or early translational activity in other immune-mediated diseases, and experimental studies in selected hepatobiliary disorders suggest that these approaches can alter the Th17/Treg balance. By contrast, therapeutic manipulation of the Tfh–B-cell–IgA axis remains largely confined to mechanistic investigation. Importantly, such downstream immune interventions would not directly correct upstream microbial or metabolic abnormalities, while systemic immune modulation may carry risks including infection, excessive immunosuppression, and non-specific disruption of host–microbiota homeostasis.

Overall, candidate strategies related to the mechanistic framework proposed in this review currently span markedly different levels of evidentiary maturity. Bile acid-directed interventions are supported by relatively extensive clinical and translational evidence from hepatobiliary diseases, whereas microbiota remodeling, targeting of microbial metabolic enzymes, and manipulation of specific metabolic pathways rely predominantly on cross-disease and preclinical evidence. Immune interventions targeting the Th17/Treg and Tfh–B-cell–IgA axes remain largely experimental or conceptual in the context of cholecystitis. At this stage, the priority should therefore not be to expand the list of candidate therapies, but rather to identify which metabolic–immune interactions show reproducible disease relevance and causal involvement in cholecystitis.

## 5 Future perspectives

Future research should first address the current lack of cholecystitis-specific causal evidence. Across distinct clinical and pathological contexts, including gallstone-associated disease, acute cholecystitis, and chronic cholecystitis, integrated analyses combining fecal metagenomics, serum and bile metabolomics, and single-cell and spatial profiling of gallbladder tissue are needed to define the relationships among microbial functional features, metabolite exposure, and local T- and B-cell responses. In particular, the actual concentrations of candidate bile acids, SCFAs, and tryptophan-related metabolites should be quantified directly in bile and gallbladder tissues and interpreted together with TCR and BCR clonotype profiles and local immune phenotypes. Such approaches will be essential for distinguishing systemic metabolic alterations from changes in the biliary environment and from immune effects occurring locally within the gallbladder.

Building on these observations, continuous causal relationships should be established through experimentally defined microbial colonization, targeted manipulation of microbial metabolic enzymes, metabolite supplementation or blockade, and immune cell-specific interventions. Particular attention should be paid to how metabolite concentration, receptor expression, target cell identity, and the local tissue microenvironment determine the direction and magnitude of immune responses. Only after these mechanisms have been validated in cholecystitis-specific experimental models and patient cohorts will it be possible to identify metabolite–immune biomarkers with robust biological relevance and to assess their practical value for patient stratification, prediction of therapeutic response, and precision intervention.
